# 肺母细胞瘤长期追踪1例报道并文献复习

**DOI:** 10.3779/j.issn.1009-3419.2013.07.11

**Published:** 2013-07-20

**Authors:** 志明 冒, 晓华 顾, 健 孙, 网山 冒, 东兵 朱, 太峰 石

**Affiliations:** 1 226500 如皋，如皋市博爱医院胸外科 Department of Thoracic Surgery, Rugao Boai Hospital, Rugao 226500, China; 2 226500 如皋，如皋市博爱医院影像科 Department of Pathology, Rugao People's Hospital, Rugao 226500, China; 3 226500 如皋，如皋市人民医院病理科 Department of Radiology, Rugao Boai Hospital, Rugao 226500, China

肺母细胞瘤（pulmonary blastoma, PB）是一种罕见的原发于肺的恶性肿瘤，占肺原发性恶性肿瘤的0.25%-0.5%^[1]^。本文结合文献复习报道1例PB病例，初期被误诊为“右上肺良性瘤”，动态观察15个月后又被误诊为“晚期中央型肺癌”、险些失去治疗时机，行右全肺切除+淋巴结廓清手术之后无瘤生存已8年。

## 1 临床资料

患者，男，54岁，职员。2003年11月，健康体检时CT发现右上叶近肺门处2.6 cm×3.1 cm软组织密度灶，形态尚规则、密度均匀，轮廓光整、无毛刺、与肺门处大血管境界清晰，CT值23 Hu（图 1），影像学诊断为右上肺良性瘤。纤维支气管镜检查无特殊，相关检查排除结核。因无自觉症状，患者拒绝肺穿刺活检或手术，每3个月-6个月临床随访1次和CT追踪检查，病情无进展。

**1:**
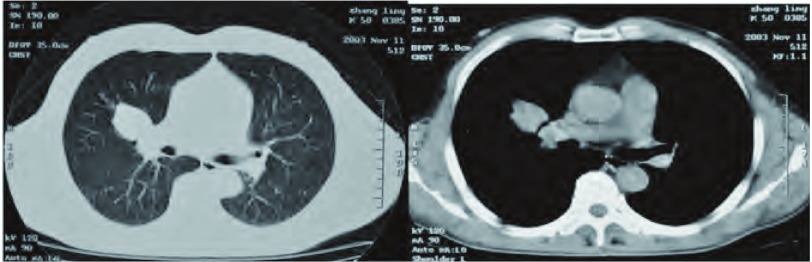
CT检查（2003年11月）：右上叶近肺门处软组织密度灶2.6 cm×3.1 cm，形态尚规则，密度均匀，边缘光整、无毛刺，与肺门、大血管境界清晰，CT值23 Hu。 CT scan of the chest (November, 2003): A tumor size is 2.6 cm×3.1 cm at superior lobe close the hilum of right lung but the boundaries between the big vessels of the gate is clear, the regular margin of the neoplasm could be observed without burr and the density is uniform, CT value is of 23 Hu.

2005年2月，患者因劳动时出现咳嗽、痰中带血就诊。CT检查示右上叶肺周边部软组织影，大小为9.5 cm×12.8 cm，分叶状、部分短毛刺，外侧缘贴近壁层胸膜，内侧缘与肺门结构分界不清，平均CT值24 Hu（图 2），影像学诊断为中央型晚期肺癌。纤维支气管镜检查：右支气管开口处轻度狭窄、腔内光滑无新生物，刷检阴性。1周后，应患者要求剖胸探查。术中见右上肺实质性肿块与壁层胸膜、肺门、心包粘连，锐性清除肺门、隆突下淋巴结，行右全肺切除。巨检显示右上肺包膜下浅灰白色肿块10.1 cm×13.2 cm，质脆嫩、易碎，剖面上见部分包膜，并有少部分壁层胸膜附着（图 3A）; 镜下显示恶性异形小细胞腺样排列，间质以梭形、原始间叶细胞为主，呈散在性分布，部分向软骨方向分化（图 3B，图 3C）; 免疫组化为Vimentin（++）、EMA（+）; 病理诊断为经典肺母细胞瘤。

**2:**
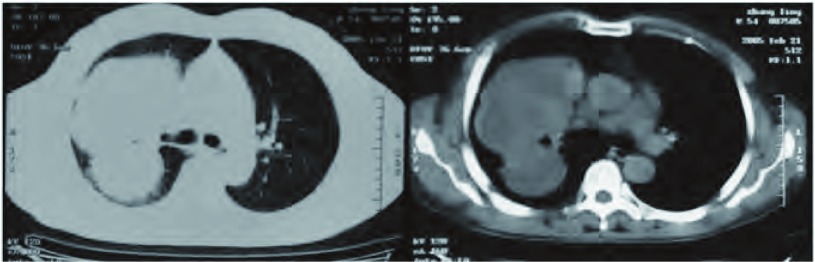
CT检查（2005年2月）：右上叶肺周边部软组织影9.5 cm×12.8 cm，分叶状、部分短毛刺，外侧缘贴近壁层胸膜，内侧缘与肺门结构分界不清，平均CT值24 Hu。 CT scan of the chest (February, 2005): A tumor size is 9.5 cm×12.8 cm at the superior lobe, its lateral edge closes the pleura and the adhesion to the chest wall is seen, the inside edge closes to the hilum of right lung and the boundary is not clear, the lobulated and blurry verge of the neoplasm could be observed with short burr, CT value is of 24 Hu.

**3:**
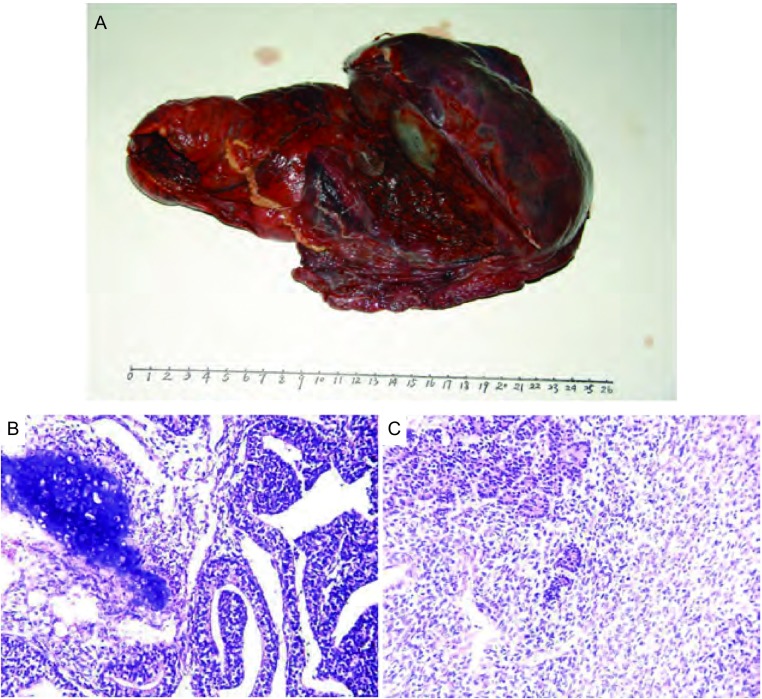
病理诊断：经典肺母细胞瘤（双向性）。A：巨检：右上叶肺包膜下浅灰白色肿块10.1 cm×13.2 cm，质脆嫩、易碎, 剖面上见部分包膜、并有少部分壁层胸膜附着; B、C：恶性异形上皮细胞呈腺样排列，间质细胞以梭形、原始间叶细胞为主，呈散在性分布，部分向软骨方向分化（HE, ×200）。 Pathological diagnosis: a pulmonary blastoma (biphase-type). A: The tumor is solitary within the lung and clinging to the visceral pleura where encroachment of a part of parietal pleura occurred and with fragile and grayish white cross section. The maximum tumor size is 10.1 cm×13.2 cm. B, C: Microphotograph is showing the malignant mesenchymal cells presented with a dispersed distribution being given priority to spindle primitive mesenchymal cells, and there are glandular organelles formed by the malignant epithelial cells. The differentiated cartilage could be seen (HE, ×200).

术后恢复良好，未再作其它治疗。随访8年，能参加一般性体力劳动。2013年5月CT复查（图 4），未见复发或转移。

**4:**
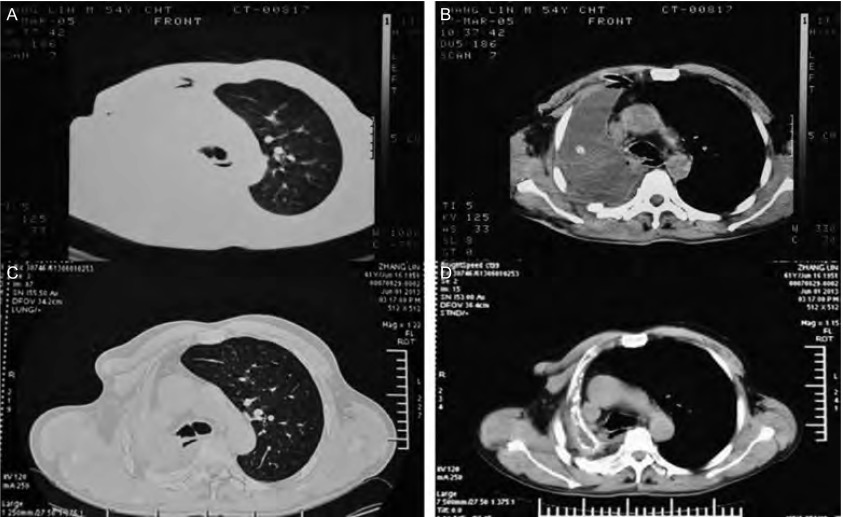
术后CT。A、B：右全肺切除术后，右侧胸腔积液、见引流管截面影（2005年3月）; C、D：纵隔右移，右侧胸廓塌陷，右全肺切除术后所见，未见肿瘤复发、转移征像（2013年5月）。 Postoperative CT scan of the chest. A, B (March 2005): After right pneumonectomy, the pleural effusion is on the right side, shadow of the chest drainage tube section is seen; C, D (May 2013): After right pneumonectomy, the mediastinum has been moved to the right with right chest collapsed. There is no expression of the tumor relapse or metastasize.

## 2 讨论

PB的组织起源尚不完全明确。Barrett和Barnard于1945年首先对这种肿瘤进行了描述、称之为“肺胚瘤”，1976年WHO将其正式命名为肺胚胎性肉瘤、又称肺母细胞瘤^[2]^。其后新分类将同时含有原始上皮及间叶成分的双向性肿瘤（经典肺母细胞瘤）、只含有原始上皮而无间叶成分的单向性肿瘤（上皮型肺母细胞瘤）和只含有原始间叶而无上皮成分的囊性或实性肉瘤（胸膜肺母细胞瘤），分别归类至肺肉瘤样癌、变异型腺癌和肺软组织肿瘤^[3]^。

依据发病年龄PB分为儿童型和成人型。儿童型为胸膜肺母细胞瘤，成人型有经典肺母细胞瘤和上皮型肺母细胞瘤两种。成人型PB各年龄段均可发生，多见于40岁-50岁年龄组，以往报道女性病例略多于或等于男性^[4, 5]^，近期报道^[6, 7]^大部分患者有长期吸烟史、男性病例多于女性。PB可以发生于任何肺叶，右肺多于左肺、上叶多于下叶。PB的影像学表现无特异性，根据部位分为周围型与中央型。以往认为绝大多数PB为周围型，位于肺周边部胸膜下，累及胸膜前无症状，累及胸膜初期仅有胸痛，出现咳嗽、喘气、咯血时已压迫或浸及肺门、支气管及大血管^[7]^;位于肺门部的中央型PB少见^[4, 5]^。近年，中央型PB明显增多、占比达30%-33.3%，体检时偶尔发现的无症状病例亦增多至25%^[6, 7]^。本例无症状时病灶邻近肺门为中央型（图 1），15个月之后出现症状时病灶增大至肺周边部胸膜下、为周围型（图 2），据此，作者认为：有必要结合PB的病期，对以往根据部位分型的方法和意义作进一步探讨。

成人型PB具有高度侵袭性，一旦出现症状后病情发展迅速、疗效差，2/3以上的病例在确诊后2年内死亡，直径 > 5 cm者术后平均生存时间为11.1个月，直径 < 5 cm者平均34个月^[7]^，但也有生存10年以上者^[5]^，关键在于早期发现、及时治疗^[7]^。事实上1/4的PB患者可在体检时偶尔发现^[6]^，如能引起重视，均可获得早期治疗。本例总结：①临床诊断“肺部良性肿瘤”应谨慎，须常规行CT引导下肺穿刺活检; ②因穿刺导致气胸等影响活检效果的近肺门处的小病灶，经短期动态观察无改观，应及时获取术中快速病理诊断、确定治疗方法^[5-7]^;③在CT追踪过程中肺良性瘤出现酷似中央型晚期肺癌的表现时，不可轻意放弃手术。本例手术结果证明，影像学表现酷似中央型晚期肺癌的PB，存在对肺门及其周围结构的挤压、粘连，有根治性切除的可能性。

扩大切除+淋巴结清扫是治疗PB的主要方式，如有淋巴结转移或周围组织受侵，可行术后放化疗。但Surmont等^[8]^报道仅少数病例对放疗敏感; Cutler等^[9]^总结术后化疗，468例患者无论单药还是联合用药效果都不理想，中位生存期仅为14.7个月。有学者^[5]^认为PB术后生存期长短，主要与切除程度相关，切除彻底者预后良好; 还有学者^[10]^认为PB手术治疗的效果与间叶成分的分化有关，肿瘤组织越幼稚、分化越单一、越类似胚胎瘤、预后越好。本例术后未进行放化疗，至今无瘤生存已8年，可能与间叶成分比较单一地向软骨方向分化有关。由于术前、术后连续长期追踪的病例报道较少，PB的诊治模式及其预后影响因素有待进一步探讨。
